# UV Inscription and Pressure Induced Long-Period Gratings through 3D Printed Amplitude Masks

**DOI:** 10.3390/s21061977

**Published:** 2021-03-11

**Authors:** Ricardo Oliveira, Liliana M. Sousa, Ana M. Rocha, Rogério Nogueira, Lúcia Bilro

**Affiliations:** Instituto de Telecomunicações, Campus Universitário de Santiago, University of Aveiro, 3810-193 Aveiro, Portugal; sousa.liliana@av.it.pt (L.M.S.); amrocha@av.it.pt (A.M.R.); rnogueira@av.it.pt (R.N.); lucia.bilro@av.it.pt (L.B.)

**Keywords:** long-period grating, additive manufacturing, UV long-period grating, pressure induced long-period grating

## Abstract

In this work, we demonstrate for the first time the capability to inscribe long-period gratings (LPGs) with UV radiation using simple and low cost amplitude masks fabricated with a consumer grade 3D printer. The spectrum obtained for a grating with 690 µm period and 38 mm length presented good quality, showing sharp resonances (i.e., 3 dB bandwidth < 3 nm), low out-of-band loss (~0.2 dB), and dip losses up to 18 dB. Furthermore, the capability to select the resonance wavelength has been demonstrated using different amplitude mask periods. The customization of the masks makes it possible to fabricate gratings with complex structures. Additionally, the simplicity in 3D printing an amplitude mask solves the problem of the lack of amplitude masks on the market and avoids the use of high resolution motorized stages, as is the case of the point-by-point technique. Finally, the 3D printed masks were also used to induce LPGs using the mechanical pressing method. Due to the better resolution of these masks compared to ones described on the state of the art, we were able to induce gratings with higher quality, such as low out-of-band loss (0.6 dB), reduced spectral ripples, and narrow bandwidths (~3 nm).

## 1. Introduction

The capability of manipulating the properties of light propagation inside an optical fiber can be achieved using different fiber optic technologies. Fiber gratings are intrinsic elements able to provide this in an elegant way, by inducing a periodic refractive index modulation along the length of an optical fiber. Long-period fiber gratings (LPGs) are one type of grating that are created by modulating the refractive index of the fiber core with periods higher than 100 μm, promoting the coupling between the propagating core mode and the co-propagating cladding modes. Their spectral response reveals attenuation bands at discrete wavelengths, each corresponding to the coupling of the core mode to a different cladding mode. The properties of LPGs have attracted considerable interest along the years, and a myriad of applications have been developed. Among them are their use as band rejection filters [1], gain flattening in erbium doped fiber amplifiers [2], to flatten, smooth, and shape the spectra of broadband sources [3,4], and in several sensor applications, including bend, strain, temperature, refractive index, etc. [5]. LPGs have been fabricated with a variety of techniques, such as irradiation through: UV laser [1], CO_2_ laser [6], and femtosecond laser [7]; electric arc discharge [8,9]; mechanically induced by pressing a periodically grooved plate on a fiber [10,11,12] using machined metallic or silica plates [10], v-grooves on a tube [13]; coil spring [14], and recently, pilling of blades [15], and 3D printing of amplitude masks [11,12]; ion implantation [16]; and periodical etching of the fiber cladding to produce a corrugated structure [17]. Among those, the CO_2_ and the UV laser inscription have been one of the most used. CO_2_ lasers are cheaper than UV lasers, and this is one of the main motivations for its use. However, deep UV lasers (e.g., krypton fluoride (KrF) at 248 nm wavelength and the argon fluoride laser (ArF) at 193 nm wavelength), are wide spread due to their wide use in optical lithography. Furthermore, through the UV inscription, it is possible to modulate the core refractive index homogeneously, contrary to side irradiation through CO_2_ laser, where additional methodologies such as fiber rotation, use of mirrors, etc., are needed [6,18,19,20]. Additionally, the widespread use of Bragg grating inscription systems using UV lasers makes its use an opportunity for the fabrication of LPGs, even considering the requirement of photosensitive fibers [5]. The fabrication of these gratings has been made by irradiating the fiber through an amplitude mask [1,7,21,22], or point-by-point (PbP) along its length [23]. While the latter technique offers flexibility to control the grating period and length, allowing us to produce different kinds of LPG profiles, it requires the use of a high resolution motorized stage, electronic shutter, and longer inscription time. Yet, the use of an amplitude mask allows for a simpler setup and operation, requiring low resolution motorized stages for scanning the beam along the fiber [7], i.e., to control the grating length, or just using the beam dimension for this effect [1,21,22]. While the first offers more control on the grating properties, both technologies are suited for mass production of multiple LPGs. Literature review on the types of amplitude masks reported so far shows the existence of four types, namely: Chrome-on-silica masks [1], patterned mirror (dielectric masks) [22], metal masks [4,5,23], e.g., Cu vapor laser milling of a copper foil [5], and microlens array [24]. Among those described above, the ones based on chrome-on-silica masks have been the most used by the research community. However, this type of mask has power limitations due to its low damage threshold. Therefore, long writing times are needed. The same also applies for dielectric masks. Furthermore, the production of these masks involves high costs. The fabrication of a microlens array-based amplitude mask is cost effective, simple, and at the same time provides high efficiency due to its superior transmission compared to conventional amplitude masks. However, the periodicity of the grating requires precision on the focal distance between the microlens array and the fiber, which can compromise the repeatability of the inscription process. Additionally, the period range is limited. Finally, metal masks have been pointed as the preferred choice for the fabrication of LPGs through the amplitude mask method, thanks to its high intensity damage threshold, making them suitable for fast inscription of LPGs, while keeping its robustness. Nonetheless, the fabrication process of such masks requires precision laser milling systems capable to drill patterns of holes in thin metallic sheets [24]. Such an approach is very optimistic, but the cost of making multiple metal masks with specific grating periods could be prohibitive. Moreover, metal masks are easy to oxidize and deform.

Additive manufacturing technologies, which consist on printing successive layers of material on top of each other to create a 3D object, hold the potential to efficiently aid and also produce devices at an economy scale. Since its emergence in 1986 [25], the technology has grown in popularity due to its opportunities and due to the efforts taken for the development of new materials and techniques. Nowadays, different materials, including polymers, metals, and ceramics could be 3D printed in a variety of ways. Among the different 3D printing technologies, the ones capable of processing polymer materials have been the preferred choice due to its low cost. Filament extrusion or melt-type techniques, such as fused deposition modelling (FDM) of thermoplastic materials, are the most popular. Its use has already been shown for the fabrication of optical components, such as fibers [26], lenses [27], photonic bridges [28], etc. Furthermore, its use has also been reported for the fabrication of a loss-tunable LPG, by mechanically pressing a periodically grooved 3D printed plate onto an optical fiber [11]. This study opened another route for inducing LPGs simply and inexpensively. However, the transmission spectra induced by such structures revealed that the process was still in the early days and needed improvements to enhance its quality. Some of those aspects may point to the existence of spectral ripples, high out-of-band loss, and low finesse. One of the drawbacks associated to FDM techniques is the intrinsic low resolution (for the work reported in [11], it was about 200 μm). As the authors agree, the coarse resolution of the 3D printer had a detrimental effect on the quality of the induced spectrum. Moreover, the FDM technology has other weaknesses, such as weak layer adhesion and long printing times, that are proportional to the printing volume and resolution of the object. Alternatively, light-based technologies, which include the stereolithography (SLA) and the digital light processing (DLP), are more attractive. In these techniques the 3D object is printed layer by layer in a building platform that moves upwards. For each layer, the liquid resin contained in a tank is polymerized with the desired shape, either through an ultraviolet laser assisted technology or by an LED screen, respectively.

Compared to the FDM technology, these light based techniques have the ability to radically improve the printing resolution (~50 μm for DLP and 1 μm for the SLA), the speed of processing and the layer to layer adhesion (making the 3D printed object mechanically more robust). While the SLA technology offers better resolution, 3D printers using the DLP technique are faster and much cheaper, with prices on the market starting from 100$ for standard DLP printers [29], which is twenty times lower than an SLA 3D printer.

In this work, amplitude masks will be fabricated using a DLP consumer grade 3D printer. These masks will be used for the first time, to inscribe permanent LPGs by the UV inscription through the amplitude mask technique. The grating growth behavior and the associated mode coupling will be analyzed, and the capability to select the location of the resonance bands will be easily accomplished through the use of different amplitude mask periods. Furthermore, the higher quality of the 3D printed amplitude masks produced in this work, compared to the ones found on state of the art for the pressure induced LPG fabrication method, will reveal gratings with better spectral characteristics, presenting low out-of-band loss, low finesse and reduced spectral ripples.

## 2. Materials and Methods

### 2.1. 3D Printed Amplitude Mask Fabrication

**Figure 1:**
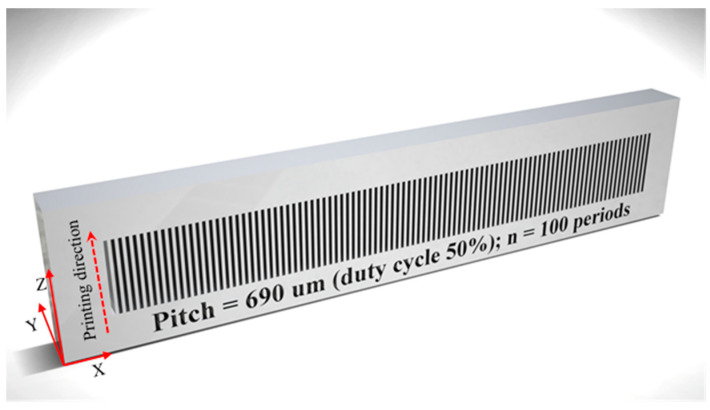
CAD (computer aided design) image of the 690 µm amplitude mask.

The fabrication of the amplitude masks started by drawing them through the help of a computer aided design (CAD) software. The masks were designed to contain 100 rectangular periods, where the duty cycle was set to 50%. Both parameters can be easily changed by the designer. However, the values presented in this work are just used as proof of concept. The mask thickness was set to 5 mm, and the height was 16 mm. The length of the mask was given by the number of periods times the grating period, plus an additional length (5 mm) at both ends of the mask, to provide robustness. To show the coupling efficiency for a wide range of wavelengths, nine amplitude masks with periods ranging from 690 to 950 μm were drawn. The corresponding CAD image of one of those amplitude masks is shown in Figure 1.

The 3D files were imported to a low cost, consumer grade, DLP 3D printer (Photon 3D printer from Anycubic, Shenzhen, China), in which the resin vat had been filled with a low cost standard colored UV resin from Anycubic (~$30 per liter) [29]. This photosensitive resin provides high-speed solidification (5 to 15 s per layer). Additionally, it provides high success rate of printing and precision. The printed materials show to be rigid and tough, presenting hardness (D) and tensile strength values of 79 and 23.4 MPa, respectively. All these parameters make this resin well suited for the purpose of this work. The 3D printer has an LCD with a resolution of 47 μm in both X and Y directions and 1 μm resolution in the Z direction. It is based on the LCD shallow masking using a 405 nm LED. The masks were printed in layers of 10 μm using an exposure time of 5 s per layer, which is sufficient to print the model with enough details. An additional exposure time of 120 s is used to print two bottom layers needed to firmly attach the model to the building platform. Ideally the amplitude mask should be printed on its longitudinal direction and with layer thickness set to 1 µm, allowing to reproduce the amplitude masks with good resolution. Unfortunately, in such configuration the liquid resin that fills the hollow regions of the mask is easily hardened by the background UV illumination, making it unsuited for the proposed application. Taking into consideration this limitation, we decided to print the masks with the printing direction indicated by the arrow shown in Figure 1. For the above parameters and taking into account the printing direction, the fabrication time was approximately 2 h. Shorter times can be achieved by reducing the exposure and/or increasing the layer thickness, however, a lower definition on the final 3D printed object will be attained. The printed masks were washed during 30 s with isopropyl alcohol in an ultrasound bath to remove the unpolymerized resin clogged into the patterned holes. They were then dried by blowing air and post-cured for 5 min using a 365 nm hand-held UV light source (Opticure LED200 from Norland Products Inc., NJ, USA), with a power density of 2.5 W/cm^2^. Finally, to increase the strength of the masks, they were thermally cured in an oven at 60 °C and 70 °C, 12 h each. The printing volume of each mask is less than 8 mL, which translates in a cost of a few cents per printed mask. Furthermore, multiple masks may be printed at the same time, allowing mass production.

### 2.2. UV Inscription of LPGs through 3D Printed Amplitude Masks

The LPG inscription through UV radiation is made with a KrF Bragg Star TM Industrial-LN excimer laser (from Coherent^®^, San Jose, CA, USA), operating at 248 nm. The laser beam exit has 6 mm in width and 1.5 mm in height, a pulse duration of 15 ns and peak power of 270 kW. The fiber is a single mode germanium doped core fiber (GF1, from Nufern^®^, Thorlabs, NJ, USA). It has a cutoff wavelength of 1260 ± 75 nm, a diameter of 9 μm for the core and 125 μm for the cladding, presenting refractive indices at the 1550 nm region of 1.4485 and 1.4440, respectively.

**Figure 2:**
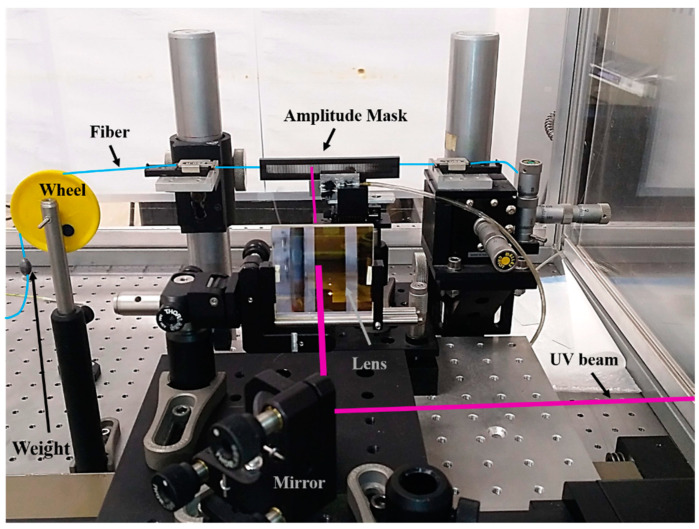
Picture of the setup used for the UV inscription of long-period gratings (LPGs) through the amplitude mask technique. The laser beam is guided through mirrors, focused by a cylindrical lens, passing through the 3D printed amplitude mask and hitting the optical fiber behind. The weight on the fiber is used to keep the same fiber strain between experiments.

The inscription is made through the amplitude mask technique, where the laser beam scans the 3D printed amplitude mask, creating a periodical pattern of illuminated-shadow regions in the core of the photosensitive fiber placed right after the amplitude mask. The efficiency of the process is further enhanced by focusing the UV beam onto the length of the fiber using a plano-convex lens. The experimental setup used for the UV LPG inscription is shown in Figure 2.

The LPGs were inscribed at a repetition rate (R) of 500 Hz and with pulse energy (E) of 3.9 mJ. A mirror placed on top of a motorized linear stage is used to scan the laser beam along the mask/fiber length, being its velocity set to 125 μm/s. The experiments were conducted by online recording the transmission spectra, where the radiation of a supercontinuum broadband optical source (Fianium Whitelase model SC-400-2, Southampton, UK) is injected into the optical fiber, and an optical spectrum analyzer (OSA) (Q8384 from Advantest, Tokyo, Japan) is used to collect the spectra. For a larger wavelength span, it was used another OSA (AQ6375 from Yokogawa, Tokyo, Japan).

### 2.3. Pressure Induced LPGs through 3D Printed Amplitude Masks

**Figure 3:**
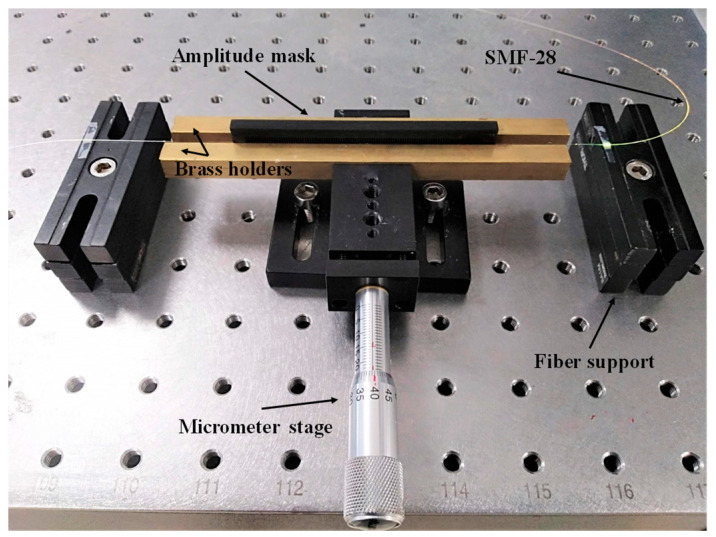
Setup used to mechanically induce LPGs in a standard optical fiber.

The mechanical pressing method is a classical method used to induce LPGs by pressing a grooved mask against an optical fiber. In this type of grating, the refractive index modulation is mechanically induced when a load is applied and disappears right after its removal. In order to show the utility of the 3D printed amplitude masks for the fabrication of LPGs using this method, they were used as the mechanical grooved plate by pressing them against a standard uncoated single mode optical fiber. The pressure was induced through a micrometer stage, which adjusts the force applied from the mask to the optical fiber. The setup used for the pressure induced method may be seen in Figure 3.

By applying displacements in the micrometer stage, it will promote the modulation of the refractive index of the fiber through the strain optic effect, which induces attenuation bands in the transmission spectra.

## 3. Experimental Results

**Figure 4:**
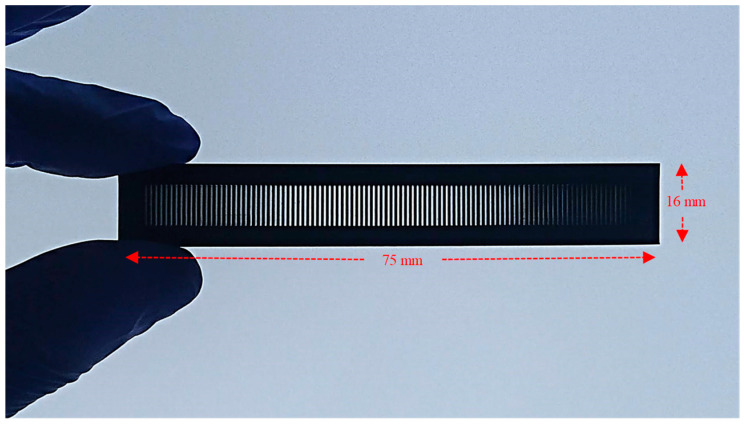
Picture of the 3D printed amplitude mask.

**Figure 5:**
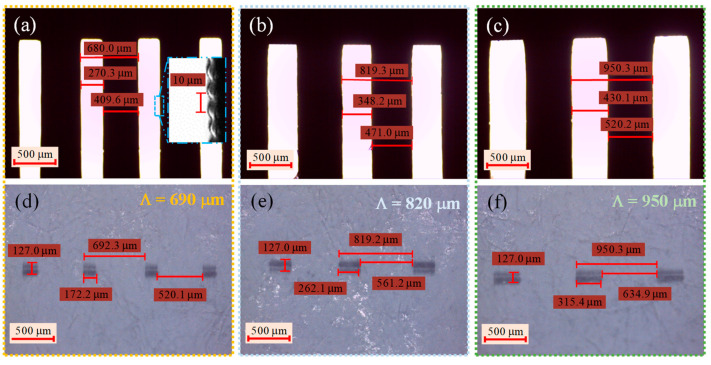
Microscope images of the amplitude masks (**a**–**c**), and the UV pattern imprinted in a thermal paper using those masks (**d**–**f**). Top images correspond to the periods of: 690 μm (**a**,**d**), middle images to the 820 μm (**b**,**e**), and bottom images to the 950 μm (**c**,**f**).

The 3D printed amplitude masks (see Figure 4) were observed under a microscope (see the images shown on Figure 5a–c) to qualitatively analyze their surface roughness and to verify if the dimensions matched the ones drawn on the CAD files.

From the inset shown in the microscope image presented in Figure 5a, it is possible to see that the edges of the grating are formed with a 10 µm periodic pattern, associated to the printing layer thickness. To achieve a smoother surface, one can adjust the 3D printer layer thickness to 1 μm, at a cost of increasing printing time. Furthermore, we tested some of those detailed masks during grating inscription and their spectral profile didn’t achieve better results compared to the ones presented in this work.

Measurements taken on the microscope images, revealed that grating period reproduces the ones predicted by the CAD files, which were ~680, 819, and 950 μm for the masks drawn with periods of 690, 820, and 950 µm, respectively. These differences are associated with the X and Y resolution and the parameters selected on the 3D printer, such as layer thickness and exposure time. Additionally, scattering effects during the UV exposure, polymer shrinkage during polymerization, resin type, and the thermal annealing could also contribute to these discrepancies. To compensate for such deviations, it is possible to adjust the printer parameters or resize the 3D model on the regions were the discrepancies are more noticeable. Yet, this was not the focus of the work, and the printed masks still reproduce the main features of the designed ones.

### 3.1. UV Inscription of LPGs with 3D Printed Amplitude Masks

We started by analyzing the UV pattern that will be imprinted in the photosensitive fiber core. To do that, a thermal paper was placed right after the amplitude mask and the laser beam was scanned along its length. The results obtained for the burnt pattern for the 690, 820 and 950 μm amplitude masks periods are displayed in Figure 5d–f. As can be observed, the transversal dimension of the focused laser beam is about 127 μm, which guaranties the full coverage of the fiber diameter. Furthermore, the periodicities of the masks are well reproduced, compared to the ones measured on the 3D printed amplitude masks shown in Figure 5a–c. The slight discrepancies were mainly regarded to the non-perpendicular orientation of the amplitude mask related to the incident UV laser beam, creating illuminated UV regions smaller than the ones presented by the mask.

**Figure 6:**
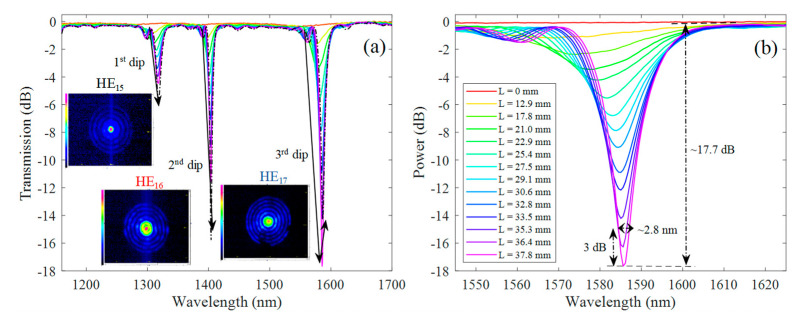
(**a**) LPG transmission spectra over time (i.e., as the grating length increases), obtained using the amplitude mask technique, by scanning a UV laser beam with velocity of 125 μm/s along a 690 μm period amplitude mask. The inset shows the experimental near-field profile of the modes corresponding to the attenuation dips. (**b**) Inset of the 3rd transmission dip (HE_17_ mode) as function of the grating length.

By scanning the laser beam through the 690 μm period amplitude mask and replacing the thermal paper by the photosensitive fiber, it was possible to obtain the results shown in Figure 6, regarding the grating transmission spectra as function of its length. The associated modes for each attenuation band were also experimentally measured and are shown as insets in Figure 6a.

**Figure 7:**
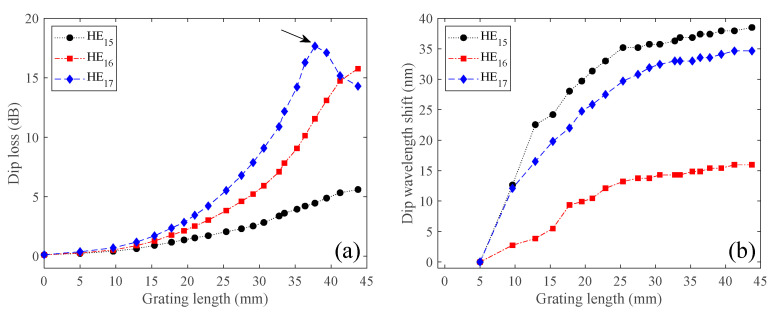
(**a**) Dip attenuation and (**b**) dip wavelength shift as function of the grating length, obtained for the LPG grating spectra shown in Figure 6.

It is worth mentioning that higher scanning velocities would be more appropriate for the reduction of the inscription time. However, the cumulative energy for each grating period is lower, and in our optimization process, we found that gratings produced with higher scanning velocities didn’t reach maximum coupling strength along the total grating length. The dip power and dip wavelength shift of each resonance band that appears in Figure 6 were measured and plotted as function of the grating length as is shown in Figure 7a,b, respectively.

As can be seen in Figure 6, the attenuation bands started to appear at 1280.4, 1388.7, and 1552.6 nm with negligible dip loss at the very beginning (see Figure 7a). Then, as the grating length increases, the resonances become stronger. The attenuation dips are also red shifted as the grating length increases (see Figure 6b). Looking to literature, both red- and blue-wavelength shifts may be observed, depending on the slope of the matching curve and more directly with the waveguide dispersion characteristics of the modes [30]. At around 38 mm (~55 periods), the deepest attenuation band (i.e., 3rd dip) reached its maximum with about ~18 dB and 3 dB bandwidth of ~3 nm (see Figure 6b), and then started to decrease (see Figure 7b). As the grating length continues to increase, the same will also occur for the two other dip resonances due to the oscillatory behavior of the LPGs, i.e., the power oscillates periodically between the fundamental core mode to the cladding mode along the LPG length. However, we stopped the process when the grating reached about 45 mm. For this length, the spectra had negligible out-of-band loss (~0.2 dB), showing resonance wavelengths at 1318.9, 1404.7, and 1587.3 nm, with dip losses of 5.6, 15.8, and 14.3 dB, and 3 dB bandwidths of 10.1, 1.9, and 3.7 nm, associated to the 1st, 2nd, and 3rd dip resonances, respectively. Taking into account that the laser scanning velocity was 125 μm/s, it is possible to estimate a total time of 6 minutes to inscribe the LPG.

The estimated cumulative number of pulses per grating period, considering R = 500 Hz and beam width of 8 mm (i.e., taking into account the beam divergence after 1 m), was 32,000 pulses. Considering the 3.9 mJ per pulse, this transduces to an estimated total cumulative energy in the photosensitive core region (i.e., just 9 μm of the 127 μm transversal focused beam width) of ~8.8 J. We also estimated the refractive index change by inscribing a fiber Bragg grating in an unperturbed region of the fiber. This has been accomplished by measuring the Bragg wavelength shift during UV inscription [31], achieving a value of approximately 1 to 2 × 10^−3^.

In order to know the excited cladding modes, the near-field profile was measured and is shown on the inset of Figure 6a. As can be seen, the fundamental core mode (HE_11_) has coupled to circularly symmetric cladding modes, i.e., HE_15_, HE_16_, and HE_17_, corresponding to the 1st, 2nd, and 3rd attenuation dips in the transmission spectra. The result was expected, since the refractive index perturbation created by the UV irradiation is evenly distributed in the fiber core, allowing us to preferentially excite circularly symmetric cladding modes [32].

**Figure 8:**
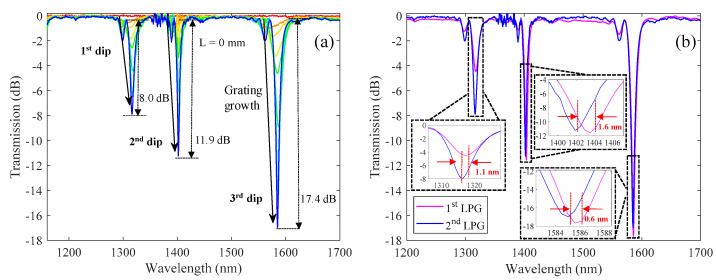
(**a**) Transmission spectra acquired during the grating inscription, for an LPG written with the same conditions as the one presented in Figure 6, (i.e., scanning the UV laser beam (E = 3.9 mJ, R = 500 Hz), with velocity of 125 µm/s along a 690 µm period amplitude mask). (**b**) Transmission spectra of two ~38 mm LPGs, either from the results collected in Figure 6 (1st LPG) and for a new grating with similar writing parameters (2nd LPG).

In order to show the reproducibility of the technique, the inscription of a new LPG (2nd LPG) was performed, using the same amplitude mask period, fiber, and laser parameters. The grating transmission spectra as function of its length for this new grating is shown in Figure 8a. For comparison purposes, the spectrum reached for a total length of ~38 mm for 2nd LPG was plotted together with the one obtained in Figure 6 (1st LPG), for the same grating length. The results may be seen in Figure 8b.

As is observed in Figure 8a, the new grating (2nd LPG) presents similar characteristics as the one shown in Figure 6a, showing three dip resonances located in the same spectral region, and presenting an increase of their coupling strength with increasing grating length. From Figure 8b, it is possible to observe that the two gratings are superimposed to each other, showing few discrepancies in between. To make a quantitative analysis, we measured the spectral parameters obtained for each spectrum shown in Figure 8b. The results may be seen in Table 1.

From Table 1, we may observe that the location of the dip resonances for each of the two gratings is similar, showing differences of about 1.1 nm, 1.6 nm, and 0.6 nm, for the 1st, 2nd, and 3rd dip resonances, respectively. The dip loss and bandwidths reached for the 2nd and 3rd dip resonances showed good agreement between the two LPGs, presenting values of 0.3 dB and ≤0.4 nm difference, respectively. Finally, on what concerns the 1st dip resonance, it was observed some discrepancies. Here, the 2nd LPG showed a dip loss with twice the value of the 1st LPG. On the other hand, the bandwidth of the 2nd LPG was half of that achieved for 1st LPG. This discrepancy was related to the fast growth of the 1st dip resonance observed in the 2nd LPG. However, since the coupling strength is a function of the grating length, it is possible to adjust this parameter in order to achieve the desired coupling strength.

**Figure 9:**
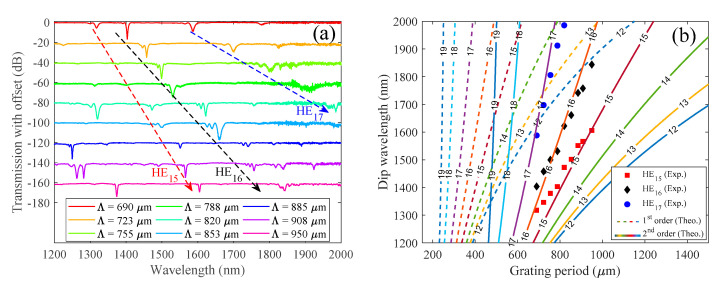
(**a**) Transmission spectra (with offset, tick spacing of 20 dB), acquired for the permanent LPGs inscribed through UV radiation by the amplitude mask technique, using different periods. (**b**) Experimental (marker points) and calculated (curve lines) resonance wavelengths as function of the grating period for the coupling of the fundamental core mode to the first eight cladding modes. The dashed and solid lines correspond to the 1st and 2nd order diffractions, respectively. The numbers in–between each line, define the subscript m, n of the HE_mn_ modes.

The use of different amplitude mask periods allows us to control the position of the resonance wavelength. One approach to change the period imposed on the fiber is by tilting the amplitude mask related to the fiber [10]. However, this technique could lead to difficulties in selecting a specific angle and/or limiting the period length due the dimensions of the mask. Due to the easiness on the fabrication of 3D amplitude masks, the capability to mass produce a specific amplitude mask grating period is easily accomplished, and this drove us to follow this path. The transmission spectra (with offset) of the LPGs produced for the different amplitude masks periods are found on Figure 9a, while the dip wavelength associated to each attenuation band is shown as a function of the grating period in Figure 9b. Note that the dip loss was optimized for the attenuation band associated to the HE_16_ cladding mode. Thus, weak power couplings for the resonances associated to the HE_15_ and HE_17_ were sometimes observed, but are still possible to track. Furthermore, it is also possible to observe that other wavelength resonances start to appear at shorter wavelengths. However, we just track the wavelength dips found for the HE_15_, HE_16_, and HE_17_ modes. As shown in Figure 9b, the dip wavelength increases as the grating length increases, showing the viability of the proposed technique for the production and control of good quality LPGs. While the loss of the resonance bands produced in this work achieved values up to 18 dB (98.4% loss), it is still possible to further improve the process by enhancing the fiber photosensitivity through hydrogen loading.

In addition, we have calculated the resonance wavelengths versus grating periods, i.e., the phase matching curves, of the HE_1n_ modes for the 1st and 2nd order diffractions. For that, the coupled mode theory was used, being the values of the electric field distribution and the effective refractive indices of the propagation modes calculated using the Wave Optics Module from Comsol Multiphysics^®^ (COMSOL, Stockholm, Sweden) software. Furthermore, it was considered a refractive index modulation with a rectangular profile and duty cycle of 40%. The value of the refractive index change was varied between the range of values estimated experimentally, i.e., 1 to 2 × 10^−3^. By using the above parameters, we found that the theoretical phase matching curves match the experimental ones for the 2nd order diffraction, and the refractive index modulation value that best fits the phase matching curves with the experimental data is 1.5 × 10^−3^. The results are shown in Figure 9b for both 1st and 2nd order diffractions.

During the UV inscription, it was observed that the amplitude mask irradiated region changed its surface appearance to dark. The phenomenon is intrinsically related to the strong absorption of polymer materials at the UV region, leading to degradation of the polymer material as well as ablation. To avoid such phenomenon, one can reduce the laser repetition rate or energy power, at the cost of longer inscription time. However, the gratings inscribed in this work had never been compromised, even considering the tens of tests made using the same mask in our earlier experiments.

The results shown in this work for the inscription of LPGs through a 3D printed amplitude mask were performed for a commercial available germanium core doped fiber. However, the technique can be used to any other optical fiber that shows photosensitivity under UV exposure. It is then worth to mention that the spectral behavior is not only related to the laser parameters and amplitude mask parameters, but also related to the type of fiber used. Thus, for each fiber type, it is necessary to do an optimization process, in order to reach the desired spectral profile. Furthermore, the grating inscription through the 3D printed amplitude mask has been performed for a specific UV laser (i.e., 248 nm). However, taking into account that polymers show strong absorption at the UV region, we don’t see any problem in using 3D printed masks to other UV laser sources, as long as the laser parameters are properly adjusted.

The periods covered in this work ranged from 690 µm up to 950 µm, allowing us to shift the resonance wavelengths almost 300 nm. From the phase matching curves observed in Figure 9, it is possible to analyze that the turning points, well known from its high sensitivity [33], are located for wavelengths longer than 2000 nm, which isn’t practical due to the low availability of sources and detectors for those regions. However, we stress out that grating periods smaller than the ones used in this work are also possible to be 3D printed with proper adjustment of the laser printer parameters. In fact, the resolution of the 3D printer is the only limitation. Thus, the inscription of 1st order gratings with turning points in suitable wavelength regions could be possible. However, this was not the focus of this work, being our main motivation the description of the possibility to inscribe gratings through simple and low cost 3D printed amplitude masks.

### 3.2. Pressure Induced LPG through 3D Printed Amplitude Masks

**Figure 10:**
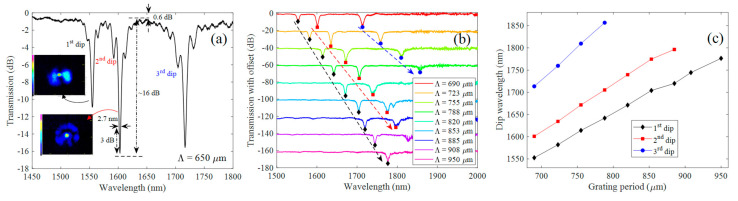
LPG transmission spectra acquired with the pressure induced method for a 3D printed amplitude mask with: (**a**) 690 μm period and (**b**) 690–950 μm periods. The insets shown in (**a**), are the experimental near-field profiles of the 1st and 2nd dip resonances. The screw displacement was set to induce the deepest mode losses. (**c**) Dip wavelength shift as function of the grating period for the different LPG resonances seen in (**b**).

The utility of the fabricated amplitude masks is not only restricted to the UV inscription of LPGs, but can also be applied to create LPG through the pressure induced method. To show this, the masks used for the UV inscription method were also used as the mechanical grooved plate for the mechanical pressing method. To do that, the masks were pressed against a standard uncoated optical fiber as shown in Figure 4. The transmission spectrum obtained for a 690 μm period mask and the ones obtained for a wide range of amplitude mask periods (i.e., 690–950 μm), may be seen in Figure 10a,b, respectively.

The spectrum shown in Figure 10a was obtained for a micrometer displacement of ~100 μm, showing three sharp dip resonances (~3 nm bandwidth at 3 dB), with dip loss reaching ~16 dB for the strongest dip resonance (i.e., 2nd transmission dip), being the resonances located at ~1555 nm, 1603 nm, and 1716 nm. Due to the viscoelastic nature of polymers, as is the case of the material that composes the 3D printed amplitude mask, the dip resonances change their coupling strength during a few tens of seconds after applying the load. This occurs due the molecular rearrangement of the polymer chains that will tend to dissipate part of the accumulated energy as plastic deformation. This process occurs up to a saturation point, that is reached after some time. Because of this property, the final spectrum was taken two minutes after applying the final load. Regarding the case observed in Figure 10a, we used a micrometer displacement of ~100 µm, obtaining a well-defined grating with ~0.6 dB of background loss. Increasing the load applied to the mask may induce stronger core to cladding couplings. However, this comes at a cost of higher background loss. Furthermore, for higher loads it is possible to fall in the plastic regime of the material, which becomes unsuited to reproduce the spectra of consecutive mechanically induced gratings.

The near-field profile of the associated mode couplings was measured for the 1st and 2nd wavelength resonances. The measurement of the 3rd dip resonance was not possible due to the absence of a laser source at this wavelength region in our laboratory. The near-field profiles may be seen in the insets of Figure 10a. Despite the symmetric profile appearance, these modes were highly dependent on the changes of the incident polarization light state, showing slight power variation on the cladding region. This is a result of the non-symmetric periodic perturbations induced on the fiber cladding that promote the coupling with antisymmetric cladding modes, leading to birefringence.

The results regarding the transmission spectra (with offset), acquired for a similar micrometer displacement as the one used for the 690 µm amplitude mask (~100 µm), for other grating periods, are shown in Figure 10b. From this figure, it may be seen that the gratings revealed a similar spectral profile as the one observed in Figure 10a, presenting dip losses reaching in some cases values up to 18 dB. Furthermore, the results showed a red-wavelength shift of the spectra with increasing grating period. By tracking the dip wavelength of the resonances, we were able to display the phase curves as shown in Figure 10c. These curves show that for the wavelength region covered in this work, there is a monotonous increase of the resonance wavelength as a function of the grating period (rate of 0.9 to 1.5 nm/μm). The results reveal that the amplitude masks are not only restricted to the inscription of UV gratings, but are also able to be used as the mechanical grooved plate in the classical pressure induced method. Regarding the latter, and taking into account the higher resolution of the DLP 3D printer used in this work, compared to that of the FDM used in [11], a smoother surface is obtained, giving more detail to the mask and promoting an associated spectrum with better optical properties, namely, reduced ripples at the background, low out-of-band loss (i.e., 0.6 dB compared to the 5 to 10 dB found in [11]) and sharp dip resonances with linewidths up to ~3 nm, which were associated to the large number of periods used (100 periods) [1]. Furthermore, the quality of the 3D printed masks allowed to linearly adjust the resonant wavelengths by changing its grating period, which is difficult to achieve through the use of low resolution printers.

In the overall, the capability to produce LPGs through simple, low cost and high resolution 3D printed amplitude masks makes the possibility of producing LPGs in simpler and effective means, promoting an opportunity for the use of this fiber optic filter more intensively in the future.

## 4. Conclusions

In this work we have demonstrated the ability to easily and inexpensively fabricate amplitude masks through a consumer grade 3D printer, allowing to create LPGs, through the UV scanning method and also by the mechanical pressing method. The results showed gratings with well-defined resonance dips, presenting negligible out-of-band loss (~0.2 dB), small 3 dB bandwidths (i.e., ~3 nm), and with dip losses up to ~18 dB. However, we stress that the control of specific grating parameters, such as bandwidth, dip loss, grating profile, wavelength resonance, etc., are functions of the laser parameters (cumulative energy per grating period) and grating parameters, such as grating profile, grating period, number of periods, duty cycle, etc. One example of this type of control was shown for the wavelength position of the resonance bands, where the flexibility in 3D printing different amplitude mask periods was used to show the possibility to shift the LPG attenuation bands. We believe that this work will pave the way for a simpler and efficient way to fabricate LPGs through the UV method, allowing them to be used in sensor or telecommunications applications.

## Figures and Tables

**Table 1:** Comparison of the grating spectral parameters for two 690 µm period LPGs written in the same conditions and with ~38 mm length.

Grating	Dip Resonance	Wavelength (nm)	Dip Loss (dB)	3 dB Bandwidth (nm)
1st LPG ^1^	1st	1317.8	4.5	15.0
	2nd	1404.1	11.6	3.2
	3rd	1586.2	17.7	2.8
2nd LPG ^2^	1st	1316.7	8.0	6.6
	2nd	1402.5	11.9	2.8
	3rd	1585.6	17.4	3.0

^1^ LPG shown in Figure 6. ^2^ LPG shown in Figure 8a.

## References

[1] Vengsarkar A.M., Lemaire P.J., Judkins J.B., Bhatia V., Erdogan T., Sipe J.E. (1996). Long-period fiber gratings as band-rejection filters. J. Lightwave Technol..

[2] Vengsarkar A.M., Bergano N.S., Davidson C.R., Pedrazzani J.R., Judkins J.B., Lemaire P.J. (1996). Long-period fiber-grating-based gain equalizers. Opt. Lett..

[3] Hodgson C.W., Vengsarkar A.M. (1996). Spectrally shaped high-power amplified spontaneous emission sources incorporating long-period gratings. Proceedings of the Optical Fiber Communications Conference.

[4] Swart P.L. (2004). Long-period grating filter with tunable attenuation for spectral equalization of erbium-doped fiber broadband light sources. Opt. Eng..

[5] James S., Tatam R. (2003). Optical fibre long-period grating sensors: Characteristics and application. Meas. Sci. Technol..

[6] Almeida T., Oliveira R., André P., Rocha A., Facão M., Nogueira R. (2017). An automated technique to inscribe reproducible long-period gratings using a CO_2_ laser splicer. Opt. Lett..

[7] Heck M., Krämer R.G., Ullsperger T., Goebel T.A., Richter D., TÜnnermann A., Nolte S. (2019). Efficient long period fiber gratings inscribed with femtosecond pulses and an amplitude mask. Opt. Lett..

[8] Almeida T., Shahpari A., Rocha A., Oliveira R., Guiomar F., Pinto A., Teixeira A., André P., Nogueira R. (2016). Experimental Demonstration of Selective Core Coupling in Multicore Fibers of a 200 Gb/s DP-16QAM Signal. Proceedings of the Optical Fiber Communication Conference.

[9] Palai P., Satyanarayan M.N., Das M., Thyagarajan K., Pal B.P. (2001). Characterization and simulation of long period gratings fabricated using electric discharge. Opt. Commun..

[10] Savin S., Digonnet M.J.F., Kino G.S., Shaw H.J. (2000). Tunable mechanically induced long-period fiber gratings. Opt. Lett..

[11] Iezzi V.L., Boisvert J.S., Loranger S., Kashyap R. (2016). 3D printed long period gratings for optical fibers. Opt. Lett..

[12] Lee J., Kim Y., Lee J.H. (2020). A 3-D-printed, temperature sensor based on mechanically-induced long period fibre gratings. J. Mod. Opt..

[13] Rego G., Fernandes J.R.A., Santos J.L., Salgado H.M., Marques P.V.S. (2003). New technique to mechanically induce long-period fibre gratings. Opt. Commun..

[14] Yokouchi T., Suzaki Y., Nakagawa K., Yamauchi M., Kimura M., Mizutani Y., Kimura S., Ejima S. (2005). Thermal tuning of mechanically induced long-period fiber grating. Appl. Opt..

[15] Torres-Gómez I., Ceballos-Herrera D.E., Salas-Alcantara K.M. (2020). Mechanically-induced long-period fiber gratings using laminated plates. Sensors.

[16] Fujimaki M., Ohki Y., Brebner J.L., Roorda S. (2000). Fabrication of long-period optical fiber gratings by use of ion implantation. Opt. Lett..

[17] Lin C.Y., Wang L.A., Chern G.W. (2001). Corrugated long-period fiber gratings as strain, torsion, and bending sensors. J. Lightwave Technol..

[18] Grubsky V., Feinberg J. (2006). Fabrication of axially symmetric long-period gratings with a carbon dioxide laser. IEEE Photonics Technol. Lett..

[19] Tian F., Kanka J., Zou B., Chiang K.S., Du H. (2013). Long-period gratings inscribed in photonic crystal fiber by symmetric CO_2_ laser irradiation. Opt. Express.

[20] Oh S.T., Han W.T., Paek U.C., Chung Y. (2004). Azimuthally symmetric long-period fiber gratings fabricated with CO_2_ laser. Microw. Opt. Technol. Lett..

[21] Lu W., Lu L., Feng F., Shi J. (2014). Low-cost amplitude mask for long-period grating fabrication. Optik.

[22] Patrick H.J., Askins C.G., McElhanon R.W., Friebele E.J. (1997). Amplitude mask patterned on an excimer laser mirror for high intensity writing of long period fibre gratings. Electron. Lett..

[23] O’Regan B.J., Nikogosyan D.N. (2011). Femtosecond UV long-period fibre grating fabrication with amplitude mask technique. Opt. Commun..

[24] Liu S.Y., Tam H.Y., Demokan M.S. (1999). Low-cost microlens array for long-period grating fabrication. Electron. Lett..

[25] Hull C.W. (1986). Apparatus for Production of Three-Dimensional Objects by Stereolithography.

[26] Chu Y., Fu X., Luo Y., Canning J., Tian Y., Cook K., Zhang J., Peng G.-D. (2019). Silica optical fiber drawn from 3D printed preforms. Opt. Lett..

[27] Berglund G.D., Tkaczyk T.S. (2019). Fabrication of optical components using a consumer-grade lithographic printer. Opt. Express.

[28] Lindenmann N., Dottermusch S., Goedecke M.L., Hoose T., Billah M.R., Onanuga T.P., Hofmann A., Freude W., Koos C. (2015). Connecting silicon photonic circuits to multicore fibers by photonic wire bonding. J. Lightwave Technol..

[29] Anycubic 3D Printing. https://www.anycubic.com.

[30] MacDougall T.W., Pilevar S., Haggans C.W., Jackson M.A. (1998). Generalized expression for the growth of long period gratings. IEEE Photonics Technol. Lett..

[31] Mizunami T., Fukuda T., Hayashi A. (2004). Fabrication and characterization of long-period-grating temperature sensors using Ge-B-co-doped photosensitive fibre and single-mode fibre. Meas. Sci. Technol..

[32] Erdogan T. (1997). Fiber grating spectra. J. Lightwave Technol..

[33] Shu X., Zhang L., Bennion I. (2001). Sensitivity characteristics near the dispersion turning points of long-period fiber gratings in B/Ge codoped fiber. Opt. Lett..

